# Advanced starch-based films for food packaging: Innovations in sustainability and functional properties

**DOI:** 10.1016/j.fochx.2025.102662

**Published:** 2025-06-17

**Authors:** Arun Karnwal, Abdur Rauf, Amar Yasser Jassim, Manickam Selvaraj, Abdel Rahman Mohammad Said Al-Tawaha, Piyush Kashyap, Deepak Kumar, Tabarak Malik

**Affiliations:** a Department of Microbiology, Graphic Era (Deemed to be University), Dehradun, Uttarakhand, India; b Department of Chemistry, University of Swabi, Swabi, KP, Pakistan; c Department of Marine Vertebrate, Marine Science Center, University of Basrah, Basrah, Iraq; d Department of Chemistry, Faculty of Science, King Khalid University, Abha 61413, Saudi Arabia; e Research Centre for Advanced Materials Science (RCAMS), King Khalid University, AlQura'a, P.O. Box 960, Abha, Saudi Arabia; f Department of Biological Sciences, Al Hussein bin Talal University, PO Box 20, Maan, Jordan; g Department of Food Technology and Nutrition, School of Agriculture, Lovely Professional University, Phagwara 144411, Punjab, India; h Department of Chemistry, Manipal University Jaipur, Jaipur, Rajasthan 303007, India; i Department of Biomedical Sciences, Institute of Health, Jimma University, Ethiopia; j Division of Research and Development, Lovely Professional University, Phagwara, Punjab, India

**Keywords:** Starch-based films, Biodegradable packaging, Food preservation, Nanotechnology, Mechanical properties, Barrier enhancements

## Abstract

Starch-based edible packaging films have emerged as a sustainable alternative to conventional plastics due to their biodegradable nature, low cost, and abundance. Recent innovations focus on overcoming limitations such as low mechanical strength, moisture sensitivity, and limited barrier properties. Incorporating nanomaterials like cellulose nanofibers and montmorillonite has significantly improved mechanical and barrier properties. Composite films combining starch with biopolymers like chitosan and PVA have enhanced flexibility and antimicrobial capabilities, making them ideal for food packaging. Advances in active and intelligent packaging are transforming these films into multifunctional solutions. For instance, films infused with natural extracts such as clove oil exhibit antimicrobial properties, while pH-sensitive indicators provide real-time food freshness monitoring. Emerging technologies like 3D printing and reactive extrusion enable tailored film designs while utilizing agricultural waste as raw material to enhance sustainability. These advancements make starch-based films pivotal in addressing environmental concerns and meet evolving consumer demands for eco-friendly packaging solutions, though challenges in industrial scalability and cost-effectiveness remain.

## Highlights

• Nanomaterials improve the strength and barriers of starch-based edible films.
• Active films with clove oil extend food shelf life by inhibiting pathogens.
• Intelligent films signal food spoilage using pH-sensitive natural dyes.
• 3D printing and reactive extrusion optimize tailored film applications.
• Agricultural waste utilization boosts cost-effectiveness and sustainability.

## 1 Introduction

**Fig. 1:**
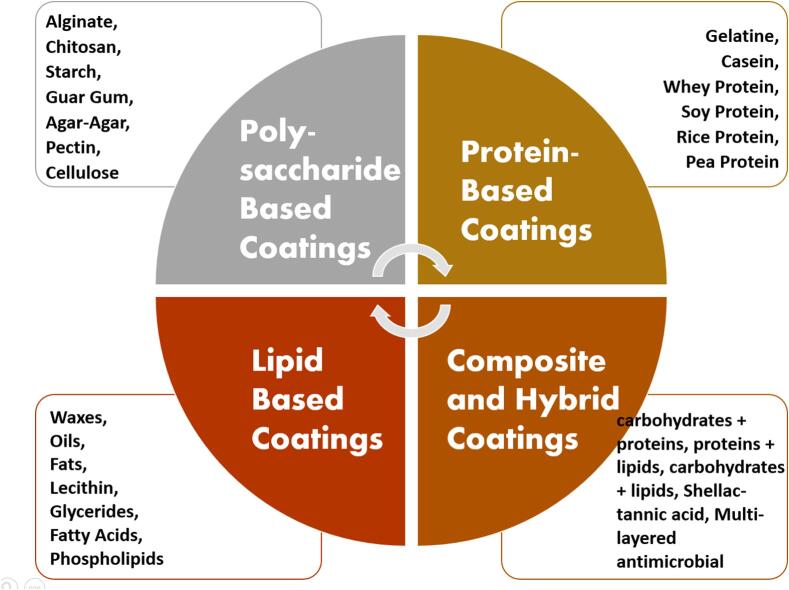
A comprehensive overview of the various categories of edible coatings materials, including polysaccharide-based, protein-based, lipid-based, and composite/hybrid coatings, highlighting their respective roles in extending the shelf life and improving the quality of food products.

Global plastic consumption has reached alarming levels, with over 368 million metric tons produced annually, contributing significantly to environmental degradation (Ayassamy, 2025; Gautam et al., 2024; Maurya et al., 2025). Approximately 79 % of this plastic waste ends up in landfills or the natural environment, leading to severe ecological consequences, including marine pollution and harm to wildlife (Jambeck et al., 2015). The urgency for biopolymer-based packaging solutions has intensified as sustainability becomes a critical focus for both consumers and corporations. According to industry reports, the global biodegradable packaging market is expected to grow at a compound annual growth rate (CAGR) of approximately 12.6 % from 2021 to 2028, reaching an estimated value of $26.3 billion by 2024. This robust growth reflects increasing demand for eco-friendly alternatives that reduce plastic pollution and align with environmental regulations worldwide. (Petrenko et al., 2024; Verma et al., 2024). Within this context, starch-based films are gaining traction due to their renewable nature, biodegradability, and versatility. These films not only offer a sustainable alternative to conventional plastics but also exhibit favorable mechanical and barrier properties, making them suitable for various packaging applications (Chatterjee et al., 2025). As industries seek to reduce their carbon footprint and comply with stricter environmental regulations, the demand for starch-based films is expected to rise, positioning them as a key player in the transition toward sustainable packaging solutions. Edible coatings have emerged as a transformative solution for preserving fresh fruits and vegetables. Extensive research has shown their ability to minimize water loss, regulate respiration rates, enhance product gloss, and inhibit microbial growth during postharvest storage. These coatings are a natural, sustainable alternative to conventional preservation methods (Hasan et al., 2020; Medeiros Silva et al., 2020; Nair et al., 2020a). However, variations in the physicochemical and mechanical properties of different biopolymers pose challenges in developing a universal, durable coating suitable for diverse food industry requirements (Biswal et al., 2024). Derived from food-grade materials such as cellulose, proteins, and polysaccharides, edible coatings are considered safe under the Generally Recognized As Safe (GRAS) status (Karnwal et al., 2025). Fig. 1 illustrates different edible coating materials (polysaccharide, protein, lipid, and composite) used in food preservation. Among these, starch biopolymers stand out due to their affordability, availability, and excellent film-forming properties (Sulistyowati, Hernani, & Supriatna, 2024). Starch-based films have useful features like being clear and colorless, having no taste, smell, or flavor, and acting as good barriers to gases like oxygen and carbon dioxide. These qualities make them a strong option for use in edible food coatings (Supplementary Fig. 1). However, their limited mechanical strength restricts industrial applications, prompting research into enhancing their durability through co-biopolymers and secondary additives (Moghadas et al., 2024). Thermal processing and post-thermal modifications further optimize their film-forming characteristics and stability over time (Sharma et al., 2021).

Developing an effective edible coating involves two key approaches: (i) material science and (ii) application to the fruit surface. The material science approach focuses on converting biopolymers into gels and transforming them into thin films, requiring an in-depth understanding of coating behavior before application (Rhim, 2004). Performance evaluation considers properties such as thickness, solubility, moisture content, water vapor permeability (WVP), oxygen barrier capacity, transparency, tensile strength, elongation at break, elastic modulus, and antimicrobial activity, all crucial for preserving produce quality and shelf life (Ghosh & Katiyar, 2022). Gelatinization and retrogradation are critical mechanisms that directly influence the performance of starch-based edible films. Gelatinization involves the disruption of starch granules upon heating in the presence of water, leading to the swelling of granules and leaching of amylose, which forms a continuous matrix essential for film formation (Sharma et al., 2021). Retrogradation, on the other hand, occurs during cooling or storage, where the linear chains of amylose and amylopectin realign and recrystallize, affecting the film's mechanical strength, barrier properties, and transparency. These transitions determine the film's flexibility, integrity, and shelf-life. Despite its significance in film formation, the impact of secondary components like plasticizers on thermal (gelatinization) and post-thermal (retrogradation) processes in starch-based edible films remains underexplored (Hasan et al., 2020; Medeiros Silva et al., 2020; Nair et al., 2020a). Plasticizers, by interacting with starch molecules, can alter water mobility and molecular interactions, thereby modulating these critical thermal transitions. A deeper understanding of these interactions is essential for tailoring film properties to meet specific packaging requirements.

These processes depend on intricate molecular interactions influencing granule swelling, amylose/amylopectin disintegration, and glass transition, all affecting film properties (Hazrati et al., 2021; Malik et al., 2022). Starch's hydrophilicity is largely influenced by its amylose and amylopectin ratio, as both contain abundant hydroxyl groups that readily form hydrogen bonds with water. Amylose, being mostly linear, tends to form stronger crystalline regions, enhancing film strength but still attracting moisture. In contrast, the highly branched amylopectin reduces crystallinity, leading to more amorphous and flexible structures but weaker mechanical properties. Overall, the abundance of hydroxyl groups promotes water absorption, which compromises the mechanical integrity of starch-based materials by disrupting hydrogen bonding networks and reducing structural stability.

Current literature primarily examines empirical changes in film characteristics rather than the underlying mechanisms of starch gelatinization and retrogradation. Understanding these interactions is essential for refining starch-based films for broader applications (Dai, Wang, et al., 2024). Starch, an abundant and cost-effective polysaccharide, is preferred for bioplastic formation due to its superior film-forming capabilities. Starches from diverse sources used alone or with other biopolymers, show promise as biodegradable packaging agents, extending the shelf life of fresh produce (Basiak & Lenart, 2013). Studies by Fakhouri et al. (2007) demonstrate the effectiveness of starch-based coatings, such as corn-starch-gelatin-sorbitol composites for preserving Red Crimson grapes, mango kernel starch coatings for tomatoes (Nawab et al. (2017), and pea starch-guar gum emulsions for Valencia oranges, reducing respiration rates and mass loss (Saberi et al. (2018). Recent advancements highlight the role of plasticizing agents and active ingredients in enhancing water barrier properties (Hazrati et al., 2021; Syafri et al., 2023; Tarique et al., 2021). Plasticizers like glycerol and mannitol improve flexibility and resilience, while natural alternatives—fatty acids, plant extracts (lotus leaf, green tea, black chokeberry, pomegranate), organic compounds (urea, sunflower oil), and essential oils—contribute antimicrobial benefits (Malik et al., 2022). The novelty of this study lies in its comprehensive exploration of how thermal (gelatinization) and post-thermal (retrogradation) transitions influence the performance of starch-based edible films, particularly in the context of underexplored factors such as the role of plasticizers and secondary additives. While much of the existing literature has focused on empirical assessments of starch film properties, this study delves into the molecular mechanisms underlying film formation, flexibility, and stability—specifically the interplay between amylose and amylopectin chains during and after gelatinization. It highlights the critical but often overlooked influence of plasticizers on these transitions, offering new insights into how film characteristics can be optimized by controlling these parameters. By addressing this knowledge gap, the study advances the design of more durable, multifunctional, and sustainable starch-based films, contributing significantly to the development of next-generation biodegradable packaging materials.

This review underscores starch-based biopolymers' potential in food packaging, focusing on overcoming limitations such as low mechanical strength, moisture sensitivity, and inadequate barrier properties. Innovations like nanomaterial incorporation, active agents, 3D printing, and reactive extrusion present promising solutions to enhance mechanical, barrier, and multifunctional properties. Starch emerges as a sustainable alternative to plastics, aligning with growing environmental demands for eco-friendly packaging solutions.

## 2 Starch: Structure and functions

**Fig. 2:**
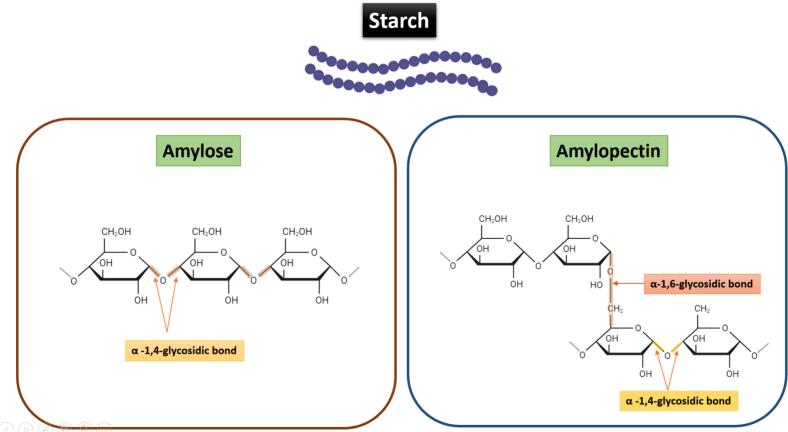
Molecular structure of amylose and amylopectin components in starch (Structure of Amylose in Starch: A linear polysaccharide composed of α-d-glucose units connected primarily by α-(1 → 4) glycosidic bonds; Structure of Amylopectin in Starch: A highly branched polysaccharide with a backbone of α-(1 → 4) glycosidic bonds and branch points linked by α-(1 → 6) glycosidic bonds).

Photosynthesis drives starch formation in plants, with glucose as the key monosaccharide. Plants absorb atmospheric CO₂, converting it into glucose, which is then polymerized into starch (Ochoa-Velasco et al., 2021). Starch consists of two polymers: amylose, a linear molecule with α-1,4-glycosidic bonds (350–1000 glucose units), and amylopectin, a highly branched polymer forming ∼70 % of starch, with α-1,6 branches every 24–30 glucose units as shown in Fig. 2 (Ghosh et al., 2021; Tester et al., 2004). Amylopectin's structure enhances solubility and enzymatic degradation. Hydroxyl groups at C-2, C-3, and C-6 confer hydrophilicity, forming hydrogen bonds that strengthen starch. However, this hydrophilicity also creates a major drawback in packaging applications. Because starch molecules readily attract and absorb water from the environment, starch-based films have poor moisture resistance. This water absorption can weaken the film's mechanical structure, increase permeability, and reduce shelf-life performance. Thus, while hydrophilicity aids in film formation and flexibility, it conflicts with the essential requirement for moisture barrier properties in food packaging materials. Upon heating, starch granules swell, burst, and undergo gelatinization, where amylose leaches out, increasing the viscosity of the solution and forming a continuous film-forming network essential for edible film development (Luo et al., 2025). Amylose enhances mechanical strength in starch-based films, whereas amylopectin dominance reduces tensile properties (Tharanathan, 2003). As a biodegradable**,** non-toxic, and edible material, starch offers a sustainable alternative to plastic, reducing environmental and health hazards in packaging (Agama-Acevedo et al., 2018). (See Fig. 1.)

**Fig. 3:**
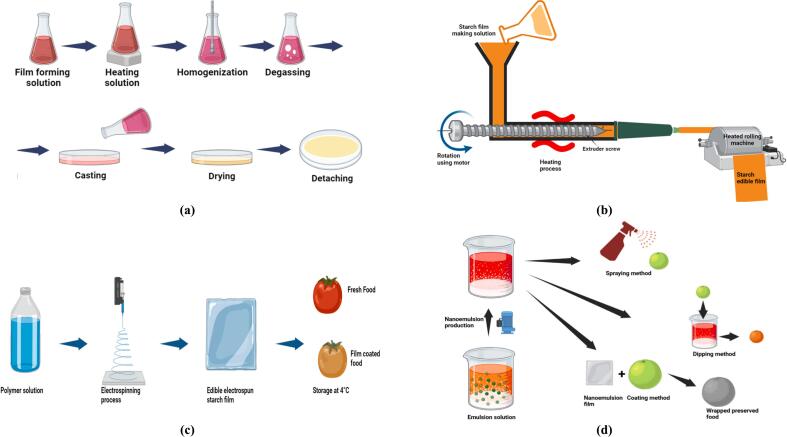
Various methods for preparing starch-based edible films: (a) *Solution casting*—starch is dissolved, gelatinized, cast, and dried into a uniform film; (b) *Extrusion*—starch, plasticizers, and additives are processed under heat and shear, then pressed into thin films; (c) *Electrospinning*—a high-voltage electric field stretches a starch solution into ultrafine nanofibers, forming a porous film; (d) *Nanotechnology-based methods*—starch is combined with nanoparticles or nanoemulsions and applied via spraying, dipping, or coating to enhance mechanical, barrier, and bioactive properties.

**Fig. 4:**
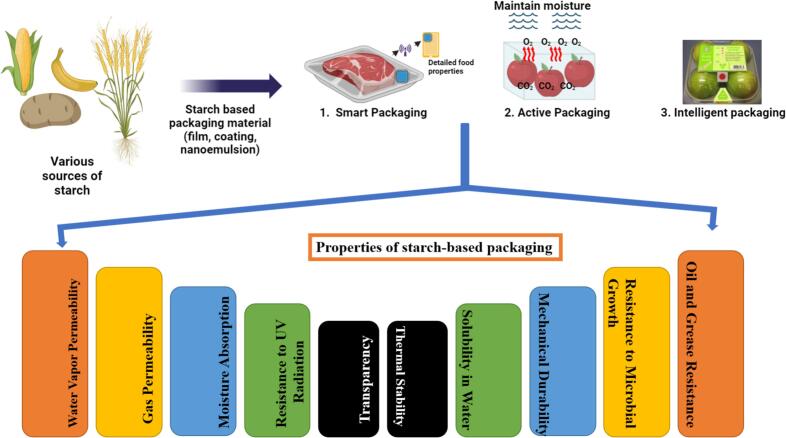
Properties and applications of starch-based biodegradable films for enhancing the shelf life of food products. These films exhibit excellent biodegradability, film-forming ability, and barrier properties against moisture and gases, making them a sustainable alternative to synthetic packaging. Their application to food products, such as fruits, vegetables, dairy, and meat, helps reduce microbial contamination, delay oxidation, and maintain freshness, extending shelf life and ensuring food safety and quality.

## 3 Gelatinization and retrogradation dynamics in starch film formation

Starch films are primarily produced through casting, where starch dispersion is applied to a smooth surface or mould and then dried (Weng et al., 2024). This method begins with heating starch granules in excess water, forming a viscous solution. The solution's instability leads to its immediate gelling upon cooling due to the natural association of polymer chains (glucose units) as they align. At elevated temperatures and with excess water, starch undergoes a crucial transition from a semicrystalline phase to an amorphous state, known as gelatinization. This transformation occurs in two distinct stages: initially, at 60–70 °C, the starch granules swell, resulting in the loss of birefringence, signaling the beginning of crystallite dissociation without a significant increase in viscosity. Above 90 °C, a second phase of swelling and solubilization occurs, causing a complete loss of structural integrity (Suhag et al., 2020).

The amylose-amylopectin ratio, water content, and dispersion temperature influence the success of this gelatinization process (Xie et al., 2024). These factors govern the recrystallization of starch during retrogradation, which significantly impacts the final properties of the starch film (Matignon & Tecante, 2017). As the water content decreases, the gel's bulk reduces until most free water evaporates, leaving behind a solid film matrix. Notably, the gelatinization temperature is directly related to the amylose content: a higher amylose concentration leads to more extensive hydrogen bonding, requiring more energy to disrupt these bonds and achieve effective gelatinization. Table 1 highlights the gelatinization behavior of various starch types as analyzed through Differential Scanning Calorimetry (DSC). It includes key parameters such as onset temperature (To), peak temperature (Tp), and gelatinization enthalpy (ΔH)**.** These values provide insights into different starch sources' thermal properties, structural characteristics, and potential applications in food and industrial processes.

**Table 1:** Various starch types and their corresponding Differential Scanning Calorimetry (DSC) gelatinization parameters.

Starch Type	Gelatinization Temperature Range (°C)	Peak Gelatinization Temperature (°C)	Enthalpy of Gelatinization (ΔH, J/g)	Remarks	Ref.
Corn Starch	60–75	67–70	6–8	Widely used in food products, moderate gelatinization temperature, high enthalpy.	(Ghoshal & Singh, 2024; Wang, Sui, et al., 2021)
Potato Starch	55–70	65–67	4–6	Lower gelatinization temperature, lower enthalpy compared to corn starch.	(Farajpour et al., 2020; Jiang et al., 2020)
Tapioca Starch	55–70	60–65	5–7	Similar to potato starch, often used for thickening and as a binder in various food products.	(Chakraborty et al., 2022)
Wheat Starch	60–75	65–70	7–9	Moderate gelatinization temperature, high enthalpy; commonly used in bakery products.	(Tao et al., 2024; Zuo et al., 2019)
Rice Starch	70–85	75–80	3–5	Higher gelatinization temperature, often used in rice-based and gluten-free products.	(Majzoobi et al., 2015; Mallick et al., 2020)
Barley Starch	60–75	67–70	5–7	Similar to corn starch but with different functional properties, used in brewing and food industries.	(Chakraborty et al., 2022)
Sweet Potato Starch	55–70	65–68	6–8	Similar to potato starch, commonly used in Asian food products and beverages.	(Jiang et al., 2020)
Arrowroot Starch	55–70	60–65	4–6	Easily digestible, often used in baby food and for thickening sauces.	(Tarique et al., 2021)
Sorghum Starch	65–80	70–75	6–8	Higher gelatinization temperature, used in gluten-free products and certain African dishes.	(Chakraborty et al., 2022)
Oat Starch	60–75	67–70	5–7	Used in gluten-free products, with mild gelatinization properties.	(Khan et al., 2024; Sulistyowati et al., 2024)
Pea Starch	55–70	60–65	4–6	Often used in plant-based food products, with similar properties to potato and tapioca starch.	(Prabha et al., 2021)
Maize Starch	60–75	65–70	6–8	Used extensively in food, pharmaceutical, and industrial applications, with good gelatinization.	(Lu et al., 2019; Wang et al., 2024)

Once the starch is gelatinized, retrogradation occurs, where dissociated amylose and amylopectin chains reassociate to form more ordered structures. This reassociation alters the starch film's permeability, solubility, and mechanical properties. The degree of crystallite disruption determines the extent of retrogradation, and this process varies with different starch compositions. Starches with higher amylose content tend to retrograde more rapidly and show increased transmittance, whereas those with higher amylopectin content undergo retrogradation more slowly due to their more significant branching and molar mass (Ploypetchara et al., 2015). The amylose-amylopectin ratio also affects the microstructure and viscosity of the starch film during heating, directly impacting the film's mechanical and barrier properties. High amylose content, while enhancing film strength, also increases its sensitivity to moisture, affecting its physical properties and overall effectiveness as a packaging material. Recent studies have highlighted various additives and approaches to mitigate retrogradation in starch films, which is crucial for enhancing their mechanical properties and stability. The incorporation of plasticizers such as glycerol and sorbitol has been shown to improve the flexibility of starch matrices, disrupting hydrogen bonding and reducing retrogradation (Zhang et al., 2020). Among the most studied plasticizers are glycerol, sorbitol, and polyethylene glycol (PEG). Each offers unique advantages and limitations when applied to starch matrices. Glycerol is the most widely used plasticizer due to its high compatibility with starch (Zhang et al., 2020). It effectively lowers the glass transition temperature, enhances chain mobility, and significantly increases the film's elongation at break. For instance, corn starch films plasticized with glycerol showed elongation rates up to 30 %, while tensile strength ranged between 2.86 and 20.64 MPa depending on starch type and formulation (Malik et al., 2022). However, glycerol's hygroscopic nature also increases moisture permeability and reduces water barrier properties, making the films less ideal for packaging high-moisture foods (Sirbu et al., 2024). Sorbitol, on the other hand, offers lower hygroscopicity than glycerol, which translates into better moisture barrier properties. Films plasticized with sorbitol typically exhibit higher tensile strength and lower water vapor permeability. For example, potato starch films with sorbitol demonstrated superior flexibility and improved film clarity compared to glycerol-plasticized counterparts. Nevertheless, sorbitol may contribute less to plasticization at low concentrations, resulting in brittler films unless carefully balanced with other additives (Hazrati et al., 2021). PEG (Polyethylene Glycol) is often used in combination with essential oils or active agents (Song et al., 2018). It provides moderate flexibility and better oxidative stability but may require higher concentrations for comparable performance. Its performance varies with molecular weight and degree of polymerization. Statistically, studies have shown that glycerol-plasticized starch films have solubility rates up to 44.76 % (corn starch), while those with sorbitol show values around 14–19.8 %, depending on starch source and treatment, confirming the lower water uptake of sorbitol-based films (Syafri et al., 2023). While glycerol excels in flexibility and ease of processing, sorbitol is superior in moisture resistance, and PEG provides a balance of durability and compatibility with active agents. The choice of plasticizer should align with the intended packaging application, considering the trade-off between mechanical performance and barrier properties. Additionally, natural polysaccharides like guar gum and xanthan gum modify the texture and stabilize starch structures, further decreasing retrogradation rates (Pal Singh et al., 2022). Enzymatic treatments using amylase have also proved effective in altering starch structures to mitigate retrogradation (Liu et al., 2019). Moreover, the development of composite films by blending starch with biopolymers or inorganic materials enhances mechanical properties while providing moisture barriers, which are essential for preventing retrogradation (Domene-López et al., 2019). These innovative approaches are vital for improving the functionality and shelf-life of starch-based films, particularly in the food packaging industry, where the ability to maintain structural integrity over time is essential (Francisco Muñoz-Gimena et al., 2023).

### 3.1 Effects of starch types on edible film properties

Starches derived from various sources exhibit a wide range of physical and chemical properties, including variations in granule shape, size, amylose-amylopectin content and ratios, and branch chain lengths (Heckl et al., 2025; Zhou et al., 2025). The performance of starch-based films is directly affected by these differences, which is why choosing the right starch for each application is essential. Starch sources, such as corn, wheat, potato, rice, tapioca, and many others, are highly diverse, each offering unique characteristics that can be leveraged for different food packaging requirements. For instance, starches from crops like quinoa, sweet potato, and millet and less conventional sources like acorns and palms offer varying film-forming capabilities (Güler et al., 2024). Table 2 provides a detailed summary of starch-based biodegradable packaging systems, highlighting the type of starch used, additives incorporated for enhanced functionality, and plasticizers to improve flexibility and durability. It also outlines the corresponding food products and applications, such as edible coatings, moisture-proof films, and antimicrobial packaging.

**Table 2:** Comprehensive Overview of Starch-Based Biodegradable Packaging Systems: Types of Starch, Additives, Plasticizers, Food Products, Applications.

Type of Starch	Additive	Plasticizer	Food Product	Application	Remarks	Ref.
Corn Starch	Cellulose nanofibers	Glycerol	Fresh Fruits	Edible coating for improved shelf life and reduced weight loss	Provides barrier to moisture and gases; enhances gloss and appearance.	(Ahari et al., 2025; Ghoshal & Singh, 2024)
Potato Starch	Chitosan	Sorbitol	Fresh Vegetables	Biodegradable packaging for moisture retention	Antimicrobial properties due to chitosan; maintains freshness.	(Farajpour et al., 2020; Malik et al., 2022)
Tapioca Starch	Nano-silica	Glycerol, Sorbitol	Bakery Products	Wrapping material to prevent moisture migration	Improved tensile strength and thermal stability.	(CS, 2022)
Rice Starch	*Aloe vera* gel	Glycerol	Meat and Poultry Products	Protective layer for microbial control and oxidation prevention	Bioactive properties reduce microbial contamination.	(Ploypetchara et al., 2015)
Wheat Starch	Essential oils (e.g., clove oil)	Polyethylene glycol (PEG)	Cheese	Antimicrobial film to inhibit mould growth	Essential oils contribute to aroma and microbial inhibition.	(Song et al., 2018)
Cassava Starch	Zinc oxide nanoparticles	Glycerol	Fish and Seafood	Active packaging to control spoilage and improve shelf life	Nanoparticles improve mechanical and antimicrobial properties.	(Lim et al., 2020)
Sweet Potato Starch	Pectin	Glycerol, Sorbitol	Ready-to-Eat Meals	Moisture-proof packaging for long-term storage	Pectin improves flexibility and water resistance.	(CS, 2022)
Corn Starch	Natural antioxidants (e.g., tea polyphenols)	Glycerol, Sorbitol	Fresh Cut Fruits and Vegetables	Extends shelf life by reducing oxidative browning and microbial growth	Combines antioxidant properties with biodegradability; suitable for fresh-cut produce.	(Ahari et al., 2025; Ghoshal & Singh, 2024)
Tapioca Starch	Silver nanoparticles	Sorbitol	Frozen Foods	Antimicrobial packaging for frozen storage	Effective at low temperatures; enhances safety and quality of frozen products.	(CS, 2022)
Rice Starch	Gelatin	Glycerol	Snack Foods	Edible film for single-serve snack packaging	Eco-friendly and enhances snack shelf life; suitable for sustainable packaging.	(Thakur et al., 2018)
Wheat Starch	Konjac glucomannan	Glycerol	Cereal Bars	Flexible wrapping to prevent moisture gain	Konjac glucomannan improves elasticity and strength.	(Song et al., 2018; Tao et al., 2024; Zuo et al., 2019)
Corn Starch	Turmeric extract	Glycerol	Spices	Antimicrobial sachets to maintain spice quality	Antimicrobial and antioxidant properties ensure longer shelf life of spices.	(Liu & Liu, 2022; Mallick et al., 2020; Wang, Sui, et al., 2021)

Granule swelling, a key step in starch processing, is significantly impacted by the granule shape, amylose-amylopectin ratio, and their interactions. Electron microscopy studies have shown that potato starch films, for example, demonstrate enhanced barrier properties, though at the expense of mechanical strength compared to films made from corn or wheat starch (Jiang et al., 2020). The thickness of films—often used to indicate permeability, optical clarity, and mechanical integrity—varies considerably across starch types. Films made from corn starch, for example, tend to be thicker (112 μm) than those derived from potato (55 μm) or wheat starch (74 μm), which directly correlates with their differing mechanical and barrier properties (Basiak et al., 2017). The solubility of starch films also varies depending on the source. High concentrations of amylopectin, for example, decrease water solubility, leading to starch granules aggregation. This aggregation can compromise the film's mechanical properties, such as strength and flexibility. Understanding the specific granule characteristics and the resulting impact on film properties is essential for optimizing starch-based packaging materials. Table 3 provides a detailed overview of various starch types combined with co-biopolymers and plasticizers to develop biodegradable films with enhanced properties. It highlights the role of heating and drying temperatures in influencing film flexibility, mechanical strength, and moisture resistance. These films are tailored for specific food product applications, ensuring improved shelf life, microbial control, and environmental sustainability.

**Table 3:** Outlining the effect of different starch types blended with co-biopolymers, plasticizers, heating, and drying temperatures on the film (Bayer, 2021; Biswal et al., 2024; Karnwal et al., 2025; Nguyen Vu & Lumdubwong, 2016; Sirbu et al., 2024; Siroha & Bangar, 2023; Syafri et al., 2023).

Starch Type	Co-biopolymer Used	Plasticizer Used	Ingredient Conc. (%)	Heating Temp. (°C)	Drying Temp. (°C)	Improved Film Properties
Corn Starch	Chitosan	Glycerol	5–10	90–100	40–50	– Increased tensile strength – Enhanced barrier properties – Better moisture resistance
Potato Starch	Gelatin	Sorbitol	3–8	80–90	50–60	– Higher flexibility – Improved film clarity – Enhanced water vapor resistance
Tapioca Starch	Pectin	Propylene glycol	4–10	85–95	45–55	– Increased film elongation – Better mechanical properties – Enhanced antioxidant activity
Wheat Starch	Xanthan gum	Glycerol	5–12	95–105	50–60	– Enhanced tensile strength – Improved elasticity – Reduced brittleness
Rice Starch	Alginate	Glycerol or Sorbitol	6–12	90–100	45–55	– Better gas barrier properties – Improved film strength and flexibility
Barley Starch	Carrageenan	Sorbitol or Glycerol	5–8	80–90	50–60	– Increased flexibility – Better water resistance – Higher opacity
Sweet Potato Starch	Gelatin	Glycerol or Sorbitol	3–6	85–95	45–50	– Improved moisture barrier – Enhanced mechanical strength – Increased transparency
Arrowroot Starch	Pectin	Glycerol or Polyethylene glycol	4–8	80–90	50–60	– Increased film durability – Enhanced water solubility – Better barrier properties
Sorghum Starch	Chitosan	Glycerol or Sorbitol	5–10	90–100	50–60	– Increased mechanical strength – Higher elasticity – Enhanced biodegradability
Oat Starch	Guar gum	Glycerol	4–7	85–95	50–60	– Improved water resistance – Better flexibility – Enhanced transparency and clarity
Pea Starch	Xanthan gum	Sorbitol	3–8	80–90	45–55	– Increased tensile strength – Improved biodegradability – Higher barrier properties
Maize Starch	CMC (Carboxymethyl cellulose)	Glycerol or Sorbitol	5–10	85–95	45–55	– Better film flexibility – Increased mechanical properties – Enhanced moisture resistance

## 4 Methods of starch film preparation

Starch's film-forming ability makes it an ideal candidate for biodegradable packaging. Unlike conventional plastics, starch is insoluble in cold water, does not melt, and has a lower degradation temperature than its melting point (Donmez et al., 2021). When exposed to force, heat, and plasticizers, starch granules lose their semi-crystalline structure, forming a flexible matrix suitable for film production. Solution casting and extrusion processing are widely used for developing high-performance, sustainable starch-based films (Donmez et al., 2021; Heckl et al., 2025; Rahmadi Putri, Adhitasari, Paramita, Endy Yulianto, & Dwi Ariyanto, 2023). Supplementary Table 1 summarizes these methods, highlighting the type of starch, food materials used, and key considerations.

### 4.1 Solution casting

Solution casting is a common technique for producing high-quality starch films (Cheng et al., 2021). The process involves three steps: (a) dissolving starch in a solvent or plasticizer, (b) casting the solution into a mould, and (c) drying to remove the solvent. Gelatinization occurs by mixing starch (3–12 %) with water and heating above its gelatinization temperature (Tgel) (Ojogbo et al., 2020a), causing granules to swell and break amylose and amylopectin chains, forming a homogeneous solution. The solution is then cast into moulds and dried using microwaves, hot-air ovens, trays, or vacuum dryers (Zhu et al., 2021). Rapid drying, however, can compromise film structure (Fig. 3a).

Water, though an effective solvent, leads to brittle films upon evaporation. Plasticizers like urea, simple sugars, sorbitol, or glycerol improve flexibility by reducing intermolecular hydrogen bonding (Hazrati et al., 2021). Solution casting offers visual appeal for packaging but requires precise water management. The method also supports antimicrobial enhancements. For instance, cassava starch films with propolis extract and cellulose nanocrystals inhibited *Staphylococcus aureus* growth in cheese for 28 days (Malik et al., 2022). Similarly, potato starch films infused with zinc oxide nanoparticles and essential oils like clove or cinnamon oil hindered bacteria such as *S. aureus, C. jejuni,* and *E. coli* (Agboluaje, 2022). Advanced modifications include nanoparticles and polymer blends like PVA, enabling intelligent packaging with colorimetric or pH indicators for real-time food monitoring (Abedi-Firoozjah et al., 2023). Starch-nano clay composites further enhance mechanical and barrier properties, making solution casting a scalable and customizable approach for eco-friendly, multifunctional films (da Silva Bruni et al., 2024; Heckl et al., 2025).

### 4.2 Extrusion process

Extrusion is preferred for commercial polymer film production due to its efficiency and ability to enhance structural and physicochemical properties. The process involves three zones: (a) feeding, (b) kneading, and (c) heating (Fig. 3b). Plasticizers such as polyethylene glycol or sorbitol (10–60 %) optimize film flexibility (Fitch-Vargas et al., 2024; Zhu et al., 2021). Mechanical and thermal energy interactions influence film quality, with screw speed adjustments affecting shear stress, uniformity, and residence time in the extruder. Higher screw speeds reduce torque, improving film properties and allowing precise adjustments with.

Stabilizers and additives. Extrusion exploits starch's thermoplastic behavior, where heating above the glass transition temperature (Tg) with reduced moisture enhances mechanical strength, elongation at break, tensile strength, and transparency (Grigsby et al., 2020). Extraction is faster and more energy-efficient than solution casting, enabling co-extrusion for multilayered films with improved functionality. For example, typical extraction processes can reduce film formation time by up to 60 %, completing in as little as 5 min compared to 12–15 min required for solution casting. Energy consumption during extraction is also significantly lower, with studies reporting reductions of 30–50 % in electrical usage due to lower drying times and elimination of solvent evaporation steps. This efficiency allows manufacturers to implement co-extrusion techniques that produce multilayer films with precise thickness control—often down to 10 μm per layer—resulting in enhanced mechanical strength and barrier properties, such as a 40 % improvement in oxygen permeability resistance compared to single-layer films made by solution casting. Extrusion also supports advanced composites. Incorporating nanoparticles like nano-ZnO and nano-SiO2 enhances film smoothness and mechanical properties (Nandhini, Bellarmin, Siva Prakash, Sowmya Sri, & Karthikeyan, 2024). Optimized extrusion conditions improve thermoplastic starch film strength and reduce retrogradation (Herniou-Julien et al., 2019). Starch-nano clay blends further enhance mechanical properties and water vapor permeability, making extrusion a cost-effective, scalable solution for sustainable packaging (Hong et al., 2022).

### 4.3 Electrospinning

Electrospinning is a versatile technique for producing ultra-fine fibers (nanometers to microns) using electrostatic interactions (Fig. 3c). Widely applied in the food and biomedical industries, it enables the development of advanced materials with unique properties (Chen et al., 2024; Muñoz-Shugulí et al., 2021). The process involves charging a polymer solution or melt with high voltage, forming a Taylor cone at the needle tip. Electrostatic forces overcome surface tension, ejecting the polymer toward a grounded collector, where the solvent evaporates, leaving solid or hollow fibers in various structures (Tan et al., 2022; Vijayakumar et al., 2023). Starch, valued for its biocompatibility, is a promising candidate for electrospinning, forming nanofibers when blended with polymers like polycaprolactone, polyvinyl alcohol, and polyglycolide (Kamoun et al., 2021). These electrospun starch fibers have applications in food packaging, drug delivery, and wound dressings (Lopez-Polo et al., 2024; Mohajeri et al., 2023). For instance, carvacrol-loaded potato starch nanofibers exhibit strong antioxidant and antibacterial properties, enhancing food preservation (Fonseca et al., 2021). Their mechanical strength and water resistance can be further improved by crosslinking with gas-phase glutaraldehyde agents (Fonseca et al., 2024). Despite its advantages, starch electrospinning faces challenges, including the need for harsh solvents like dimethyl sulfoxide (DMSO) and formic acid, raising environmental and safety concerns (Avossa et al., 2022).

Additionally, synthetic polymers, plasticizers, or crosslinking agents are often required to enhance fiber functionality. Research continues to focus on developing sustainable electrospinning techniques and improving starch-based nanofibers for broader industrial applications. Future advancements will prioritize eco-friendly processes, positioning electrospinning as a key technology for high-performance, sustainable materials.

### 4.4 3D-printing

Three-dimensional (3D) printing transforms manufacturing by enabling layer-by-layer construction of complex designs. Since its introduction by Charles W. Hull in the 1980s, it has expanded into diverse fields, including medicine, aerospace, automotive, textiles, and food (Meshram et al., 2024). In the food industry, 3D printing facilitates intricate designs, personalized nutrition, and innovative textures, offering new possibilities in food customization (Maniglia, Lima, et al., 2019). However, widespread food production adoption is limited by edible rheological and structural challenges (Feng et al., 2018). Current printable foods include dough, meat paste, cheese, and chocolate, which can be extruded into complex forms. Optimizing these materials' texture, gelation behavior, and heat resistance is crucial for expanding 3D food printing applications.

Starch has emerged as a promising gelling agent due to its superior extrudability to other starches like potato and corn (Zheng et al., 2019). Combining starch with acrylonitrile-butadiene-styrene copolymers or pea protein (Feng et al., 2018) enhances printability. Chemical modifications like ozonation improve its performance (Maniglia, Laroque, et al., 2019). As 3D printing technology advances, starch-based biodegradable food products could revolutionize the industry by addressing consumer demand for sustainable, nutritious, and customized foods while reducing packaging waste. Continued innovations in material science and printing technology will drive its future in food manufacturing.

Despite its transformative potential, 3D printing faces several limitations that hinder its broader adoption, especially in food manufacturing. One major challenge lies in the rheological and structural properties of printable materials, which must balance flowability for extrusion with stability to maintain shape post-printing (Feng et al., 2018). Many edible materials struggle with insufficient texture, weak gelation, and poor heat resistance, restricting the range of foods that can be effectively printed. Additionally, the speed of 3D printing is often slow compared to traditional mass production methods, limiting scalability for large-volume manufacturing. Equipment costs and the need for specialized printers also present economic barriers (Zheng et al., 2019). Furthermore, ensuring consistent food safety and quality in 3D-printed products requires standardized protocols, which are still under development. Addressing these technological and material challenges is critical for unlocking the full potential of 3D printing in the food industry (Maniglia, Lima, et al., 2019).

### 4.5 Nanotechnology

Nanotechnology, enabling the fabrication of materials at the 1–100 nm scale, has revolutionized material science by imparting unique properties unattainable with conventional materials. Its impact spans agriculture, food, biomedicine, and aerospace industries (Karnwal et al., 2024), addressing global challenges in health and sustainability. A key breakthrough is the development of nano-biodegradable biopolymers, which enhance agriculture, food systems, and drug delivery (Kraśniewska et al., 2020). In agriculture, nanotechnology has enabled precision tools like nano-sensors, nano-pesticides, and nano-fertilizers, improving efficiency and sustainability. The food industry benefits from nano-carriers for bioactive substances and advanced nano-packaging that extends freshness and safety (Adeyeye et al., 2019). In pharmaceuticals, nanocarriers optimize drug delivery by enabling targeted release (Karnwal et al., 2025). Among nanomaterials, starch is a key food-grade building block, with biodegradable starch-based materials emerging as alternatives to plastics (Hanan et al., 2024). Researchers have enhanced starch-based nanocomposites by incorporating nanomaterials, improving mechanical strength, crystallization kinetics, and barrier properties (Zhang et al., 2017). Fig. 3d illustrates the use of nanotechnology in preparing starch-based edible films, applying techniques like spraying, dipping, and coating to improve film properties and extend shelf life.

Nanomaterials like cellulose nanofibers boost tensile strength but can agglomerate at high concentrations (Kim et al., 2019). Montmorillonite and bentonite nanoclays enhance structural integrity, while nano‑silver and chitosan-modified bentonite provide antibacterial properties (Giannakas & Leontiou, 2017; Reis et al., 2023). The continuous evolution of nanotechnology presents vast opportunities for material innovation, sustainability, and improved quality of life.

## 5 Physicochemical properties of starch-based films

Starch, a versatile polysaccharide, has become a key material for biodegradable films due to its ability to form a continuous matrix and low oxygen permeability (Fig. 4). Starch-based films are more cost-effective than non-starch alternatives, making them attractive for sustainable packaging. However, like other hydrocolloids, starch films have limitations compared to synthetic plastics, such as their hydrophilic nature and suboptimal mechanical properties (Ilyas et al., 2024; Kupervaser et al., 2023). Starch-based films offer advantages such as transparency, odorlessness, tastelessness, and colorlessness. Their semi-crystalline structure, comprising amorphous and crystalline zones, significantly influences properties like tensile strength, gas barrier capabilities, and cohesive energy density (Lim et al., 2020). High amylopectin content in native starch increases crystallinity, but films with greater amylose content exhibit higher crystallinity due to retrogradation, where starch molecules reorganize into helices and crystal structures post-gelatinization (Luo et al., 2023; Tao et al., 2024). Starch, a versatile polysaccharide, is widely used for biodegradable films due to its ability to form continuous matrices with low oxygen permeability. However, starch films often exhibit limitations in mechanical performance compared to synthetic plastics, primarily due to their hydrophilic nature and semi-crystalline structure (Ilyas et al., 2024; Kupervaser et al., 2023). The mechanical properties of starch-based films are commonly evaluated using standard methods such as tensile strength, Young's modulus, and elongation at break, which assess film strength, stiffness, and flexibility respectively. For instance, tensile strength measurements quantify the maximum stress a film can withstand before breaking, while elongation at break indicates its ability to stretch without rupture (Hu et al., 2014). These tests reveal that potato starch films generally exhibit higher tensile strength and elongation at break compared to corn and wheat starch films, reflecting differences in molecular structure and crystallinity. Furthermore, factors like plasticizer type and concentration significantly affect these mechanical properties by modifying intermolecular interactions within the starch matrix. Thus, mechanical testing provides crucial insights into optimizing starch-based films for sustainable packaging applications (Ahmad et al., 2024; Sirbu et al., 2024). Table 4 summarizes various starch types' mechanical and physical properties, including Young's modulus, elongation at break, tensile strength, moisture content, film thickness, and solubility. Potato starch has higher tensile strength and elongation at break than other starches, while corn and wheat starches exhibit lower Young's modulus and higher solubility. Modified starches, such as oil-laminated wheat and heat-treated potato starch, demonstrate altered properties, suggesting potential for specialized applications.

**Table 4:** Physicochemical Properties and Performance of Various Starch Types in Film Formation.

Type of Starch Used	*Young Modulus* (MPa)	Elongation at Break (%)	Tensile Strength (MPa)	*Moisture Content* (%)	Film Thickness (pm)	Solubility	References
Potato	5.33	5.67	6.56	0.316	55.4	14.52	Basiak et al. [87]
Corn	0.1	19.13	3.72	0.367	112.2	44.76	
Wheat	0.12	15.21	3.29	0.445	74.1	30.16	
Wheat starch laminated with oil	0.03	16.44–18.29	0.92–1.04	2.69–2.70	22.1–27.7	7.83–10.70	
mungbean starch Wheat starch	0.08–0.10	13.18–14.16	2.03–2.10	2.01–3.24	35.4–80.8	14.49–19.67	
Topaca starch	0.8	137	0.78	17.22	136	–	Zolek-Tryznowska and Kaluza [113]
Rice starch	9.6	49	1.8	18.72	145	–	
Oat starch	1.8	27	0.36	21.77	266	–	
Potato starch	14.5	70	3.05	9.74	332	–	
Maize starch	14.2	51	1.49	22.26	266	–	
Heat and moisture treated potato starch Oxidized potato starch	7.35–24.91	38.80–84.90	6.07–9.12	–	0.102–0.124	18.89–19.89	da Rosa Zavareze et al. [115]
	8.01–8.61	79.93–84.20	6.38–7.24	–	0.118–0.147	14.78–18.87	
Potato starch	3.95–9.25	56.87–85.20	4.87–5.25	–	0.073–0.168	14.26–19.87	
Potato starch + Nanoclay (1, 2, 3 and 5 %)	297–376	44–61.5	8.09–9.82	–	–	23–30	Sadegh-Hassani, and Nafchi [102]
Potato starch	188	68	7.33	–	–	35	
OSA-modified sweet potato starch	–	–	–	13.41–14.13	0.091–098	15.25–19.72	Li et al. [101]
Sweet potato starch	–	–	–	15.2	0.106	19.99	
Cassava starch, mungbean starch, Cassava +		10.84–21.37	2.86–20.64				Vu et al. [114]
Corn and wheat	–	30	15.5	23.20–10.08	72.55–77.27	46.16–33.45	Song et al. [99]

During film formation, heating starch in water disrupts the crystalline structures of amylose and amylopectin, allowing hydration. Upon drying, these macromolecules reassociate via hydrogen bonding, impacting crystallinity. Drying and storage conditions, including temperature, relative humidity, and plasticizer presence, further influence this crystallization (Thakur et al., 2018). Plasticizers, primarily polyols, mitigate brittleness by reducing intermolecular forces within the starch matrix (Hu et al., 2014). By modifying the polymer structure, plasticizers enhance flexibility and durability. The choice and concentration of plasticizers are crucial for balancing mechanical performance and biodegradability. Plasticization primarily affects amorphous regions where molecular mobility is higher (Zhang, Su, et al., 2020). While plasticizers improve film flexibility by lowering tensile strength and increasing elongation at break, they also increase permeability to moisture, oxygen, and aroma compounds (Fonseca-Florido et al., 2019). Glycerol, a widely used plasticizer, is highly effective due to its compatibility with starch.

The mechanical properties of biopolymer films depend on plasticizer type and concentration, starch source, and storage conditions. Starch–glycerol films typically exhibit lower tensile strength and elastic modulus but higher elongation compared to starch–sorbitol films, highlighting glycerol's superior plasticizing effect (Ballesteros-Mártinez et al., 2020). Studies on other polysaccharide or protein matrices, such as soy protein (Zhao et al., 2023) and sodium caseinate (Jiménez et al., 2013), confirm this trend. Glycerol's hydrophilicity facilitates water absorption, further enhancing plasticization. However, sorbitol improves moisture barrier properties due to its lower hygroscopicity, making starch–sorbitol films better barriers than glycerol-plasticized counterparts (Sirbu et al., 2024; Ahmad et al., 2024). The glass transition temperature (Tg) dictates polymer softening and transition into a thermoplastic state. For semi-crystalline polymers, melting temperature (Tm) and crystallinity degree further influence these transitions. When Tg and Tm remain below decomposition temperature, thermoplastic processing is feasible, enabling versatile film applications (Dehghannya & Ngadi, 2024).

Storage temperatures above Tg promote recrystallization, affecting flexibility. Plasticizer molecules penetrate the polymer matrix, enhancing chain mobility, but excessive plasticizer content can cause phase separation into glycerol-rich and polysaccharide-rich zones. The critical starch–plasticizer ratio depends on the starch type and ambient humidity. Studies on cassava starch films revealed dual glass transition temperatures corresponding to distinct starch- and glycerol-rich phases (Iaccheri et al. (2023); (Meredith et al., 2024). Recrystallization significantly impacts starch films, making them susceptible to aging, where starch molecules reassociate into crystalline structures over time (Ahmad et al., 2024; Sulistyowati et al., 2024). This leads to increased strength and rigidity but reduced flexibility. Optimizing storage conditions is crucial to maintaining film functionality. Research on cassava, corn, and yam starch films with 20 % glycerol showed no significant differences in tensile strength or elastic modulus despite reduced deformability with prolonged storage, emphasizing glycerol's role in limiting crystal growth (Figueroa-Lopez et al. (2024). In contrast, glycerol-free films exhibited pronounced crystallinity increases, making them more susceptible to degradation. Variations in water vapor permeability further suggest that plasticizers stabilize film properties over time (Chang et al. (2023). These findings underscore the interplay between storage-induced crystallinity and permeability in starch-based films.

## 6 Properties of starch-based packaging

### 6.1 Barrier properties of packaging films

Barrier properties are crucial for determining the shelf life of packaged food by influencing moisture retention and protecting against microbial contamination. Effective barriers maintain food integrity, ensuring freshness and safety (Asikkutlu & Yildirim-Yalcin, 2025; Karnwal et al., 2025). Research by Iaccheri et al. (2023) indicates that carbohydrate- and protein-based coatings have limited moisture resistance due to their hydrophilicity. Hydrophobic substances, particularly lipids, enhance moisture barrier effectiveness, while surfactants improve film adhesion and resistance by reducing surface tension (Li et al., 2020). Debeaufort et al. (2022) demonstrated that hydrophobic gelatin-based plasticizers significantly enhance moisture barriers.

Water vapor permeability (WVP) is a key metric for assessing biodegradable packaging polymers. Supplementary Table 2 outlines the properties of starch-based films, including their water vapor and gas permeability, oil and grease resistance, and moisture absorption—factors influenced by composition, additives, and environmental conditions. These films are sustainable alternatives to synthetic polymers, suitable for packaging dry products, fresh produce, fatty foods, and edible films. Delassus (1997) found that mung bean starch films had high WVP (0.20–0.46 mg·mm/Pa·hr·m^2^), significantly exceeding values for high-density (0.01 mg·mm/Pa·hr·m^2^) and low-density polyethylene (0.03 mg·mm/Pa·hr·m^2^). Majzoobi et al. (2015) reported that modifying corn starch films with ethyl furan (EF) increased WVP, affecting moisture resistance. High WVP films are less suitable for commercial use as they can compromise food quality. Sharma et al. (2020) found that faba bean starch films with sodium trimetaphosphate crosslinking had lower WVP, demonstrating how starch modification enhances moisture barriers. Similarly, acetylation of rice starch films reduced WVP (Colussi et al., 2017; Mallick et al., 2020). Dual-modified banana starch showed increased WVP with oxidation but decreased permeability with acetylation (Ilyas et al., 2023).

Nanocomposite films incorporating montmorillonite or crosslinked starch (e.g., citric acid) reduce moisture sorption and swelling, enhancing barrier properties (El-Basiouny et al., 2024; Tammina et al., 2025; Zhao et al., 2025). Films incorporating carboxymethyl cellulose (CMC) and glycerol improve tensile strength and water resistance. Combinations of nano clays, xanthan gum, and cassava starch reduce WVP, enhance transparency, and maintain flexibility (Anwar et al., 2022; Iqbal et al., 2024). Oxygen vapor permeability (OVP) is another critical factor. Rompothi et al. (2017) found that mung bean starch films had lower OVP than traditional plastics. Adding glycerol (20–30 %) or sorbitol (30–40 %) further reduced OVP, making these films viable plastic alternatives. Thirathumthavorn and Thongunruan (2014) confirmed that sorbitol-plasticized mung bean starch films exhibited lower OVP than native starch films. Lipid integration significantly enhances oxygen and moisture barriers. Basiak et al. (2019) reported that laminating wheat starch films with rapeseed oil reduced OVP sevenfold while improving mechanical strength. Adding essential oils (e.g., cinnamon, ginger) slightly increased OVP but decreased WVP due to lower plasticizer content. Hydrophobic oils like rapeseed and olive oil reduce oxygen permeability by limiting water content and oxygen solubility (Hosseini et al., 2023; Tymczewska et al., 2021). Enhancing starch-based films through crosslinking, hydrophobic agents, nanocomposites, and plasticizers significantly improves barrier properties, improving shelf life and food quality retention.

### 6.2 Mechanical properties

The mechanical properties of packaging materials—tensile strength (TS), elongation at break (EAB), Young's modulus, storage modulus, and loss factor (tan δ)—are key indicators of their suitability for food packaging (Asikkutlu & Yildirim-Yalcin, 2025). These parameters determine a material's ability to withstand stress during processing, handling, and storage. TS and EAB, in particular, assess a film's resistance to breakage and structural integrity under various conditions (Guz & Famá, 2024).

Standard testing methods include texture meters, dynamic mechanical analyzers (DMA), and universal testing machines. Adding nanocellulose to starch-based films enhances tensile strength but may reduce elongation at break. DMA analysis further refines evaluation by measuring dynamic properties like storage modulus (E′) and tan δ, which indicate stiffness and glass transition temperature. The commercial viability of starch-based films depends on mechanical properties, with tensile strength being critical, alongside solubility, EAB, water vapor permeability (WVP), moisture content, and thickness. These factors determine film applications and guide manufacturers in product optimization (Karnwal et al., 2025; Zhang et al., 2023).

Nguyen Vu and Lumdubwong (2016) examined starch films from mung beans, cassava, and blends plasticized with sorbitol or glycerol. TS ranged from 2.86 to 20.64 MPa, and EAB from 10.84 to 21.37 %. Cassava starch films had lower TS due to reduced amylose content, while glycerol plasticization further decreased TS by 2 to 4 times compared to sorbitol. Basiak et al. (2016) compared wheat, corn, and potato starch films, noting that wheat starch films were more deformable and less stiff than potato starch films. Film thickness was crucial in improving TS, and water content significantly influenced mechanical properties due to starch's affinity for moisture. Lower amylose content generally correlated with increased TS and Young's modulus. Jha et al. (2020) found that corn starch films with a 28:72 amylose-to-amylopectin ratio exhibited improved TS, lower WVP, higher Tg, and better thermal stability than other starch films. Song et al. (2018) reported that a 6:4 corn-wheat starch blend had a TS of 15.50 MPa and 30 % EAB. Adding essential oils reduced TS and EAB by 28.41 % and 19.82 %, respectively, due to structural discontinuities. Basiak et al. also observed a 50 % TS reduction when incorporating rapeseed oil via lamination, confirming that oil addition weakens mechanical strength in starch-based films. Nanoparticles like nano clay, nanocellulose, and nano‑silicon dioxide significantly enhance starch film mechanics (Sadegh-Hassani & Mohammadi Nafchi, 2014). Nanoclay improves TS while maintaining or reducing EAB. Sadegh- Saberi Riseh et al. (2023) found that nanoclay increased TS in potato starch films from 7.33 to 9.82 MPa while reducing EAB from 68 % to 44 %.

Additionally, nano clay contributes antimicrobial properties, controlled release of active ingredients, and enhanced biodegradability. Studies (Bumbudsanpharoke & Ko, 2019; Hong et al., 2022; Jha et al., 2020) suggest nano clay can be used in biodegradable colorimetric indicator films for milk spoilage detection while reducing starch film solubility. Nanoparticles strengthen starch films by filling water-binding sites, reinforcing molecular interactions such as hydrogen bonding and Van der Waals forces. Xiong et al. (2019) demonstrated that nanoparticles improve wear resistance and TS. However, concerns over consumer safety and regulatory issues hinder the widespread adoption of nanocomposite films in food packaging. Despite these challenges, continued advancements in nanomaterials present promising opportunities for enhancing biodegradable packaging films.

### 6.3 Optical properties

Plasticized starch films have garnered significant interest in sustainable food packaging due to their biodegradability, transparency, and ability to protect food products. Among these, yam starch films are particularly notable for being fabricated into transparent, user-friendly films free of insoluble particles, making them suitable consumable coatings (Ghosh & Katiyar, 2022; Prabhu et al., 2021). Optical properties such as transparency and opacity are critical when designing these films because they directly affect the film's ability to protect food from light-induced spoilage while maintaining consumer appeal. For example, opacity plays a crucial role in shielding food from ultraviolet (UV) light, which can catalyze lipid oxidation and degrade sensitive nutrients. Enhancing the UV protection and opacity of starch films is often achieved through the incorporation of nanoparticles. Calcium carbonate nanoparticles incorporated into corn starch films significantly increase opacity beyond that of native starch films by scattering and absorbing incoming light (Ghosh & Katiyar, 2022). Similarly, adding talc powder to cassava starch-kaolinite composite films reduces light transmittance, improving barrier properties against harmful radiation (Ghosh & Katiyar, 2022). Nanoparticles with sizes compatible with the starch matrix integrate seamlessly, increasing opacity while maintaining the film's structural integrity. This is crucial as excessive opacity may reduce consumer acceptance due to diminished product visibility.

The structural organization within starch films also influences transparency and UV resistance. Highly structured regions reduce absorbance in the visible light spectrum, which benefits food packaging by preventing photodegradation while maintaining clarity (Ghosh & Katiyar, 2022). Plasticizers, especially glycerol, critically modulate film transparency and mechanical flexibility. However, glycerol concentration must be carefully optimized since excess amounts reduce transparency. Geleta et al. (2020) demonstrated that at 15 % glycerol concentration, starch films achieved 85 % transparency, which decreased to 81.7 % and 78.4 % at 20 % and 25 % glycerol, respectively. This trend highlights the balance needed between plasticization and optical properties. Transparency also varies according to starch source and film composition. Films prepared from botanical starches such as teff and cassava exhibit different clarity levels (Prabhu et al., 2021), and gelatin films derived from blue shark skin with 25 % glycerol show enhanced film clarity (Limpisophon et al.). Contrastingly, protein-rich films, such as those based on peanut meal, tend to be thicker and less transparent, often with a yellowish tint resulting from aldehyde-protein interactions or Maillard reactions (Cheftel et al., 1985). Adding whey protein decreases light transmission in starch and methylcellulose films, though pigments may be added to counteract oxidation in commercial products.

Blends combining glycerol, guar gum, and pea starch produce uniform and transparent films, although excessive guar gum causes phase separation, lowering clarity (Tosif et al., 2021). The addition of seed oils, like sunflower oil, reduces transparency by altering light transmittance, while chitosan integration with tapioca starch slightly improves it, with transparency values recorded at 84.7 % for native films and 85.3 % for chitosan-modified films (Li et al., 2019).

Metal oxides are especially effective in enhancing UV protection. Titanium dioxide (TiO₂) nanoparticles impart films with white color and substantial UV-blocking capabilities, compliant with safety regulations (Li et al., 2019). Even minimal concentrations of nano-TiO₂ significantly increase UV absorbance, preventing UV radiation from reaching the food product (Oleyaei et al., 2016). Likewise, nano‑silicon dioxide (SiO₂) incorporated into potato starch films improves UV resistance by decreasing light transmittance, which mitigates food spoilage caused by UV exposure (Liu et al., 2019). Cellulose nanocomposites extracted from pineapple leaves also enhance starch film transparency while providing UV protection, further demonstrating the potential of natural nanomaterials in packaging innovations (Zhang et al., 2018). These advances demonstrate the growing capability of starch-based films to balance transparency with effective UV protection, enhancing food preservation while meeting consumer demands for sustainability. The integration of nanoparticles and biopolymer composites creates films with improved optical and protective properties, paving the way for eco-friendly, functional packaging solutions.

### 6.4 Biodegradability

Biodegradability refers to the breakdown of materials into simpler compounds through microbial activity. For industries reliant on plastic packaging, biodegradability is a key concern due to environmental hazards posed by non-biodegradable polymers. The transition to eco-friendly alternatives, particularly biodegradable starch-based materials, offers a sustainable solution (Kaur et al., 2019; Leon-Bejarano et al., 2020). Biodegradable polymers have broad applications in textiles, automotive, agriculture, biomedical, and packaging sectors. Starch-based materials are gaining attention as substitutes for petroleum-derived plastics, addressing concerns over oil depletion. Various Andean crops, such as fruits, legumes, and tubers, have been explored for starch film production (Babaee et al., 2015). A comparative study (Torres et al., 2011) found cassava starch films degraded by 99.35 % in 31 days, whereas gold potato starch films degraded by 90.03 %. In contrast, cellulose films showed only a 30 % weight reduction, highlighting starch's higher susceptibility to degradation due to its glycosidic α-linkages.

Further research assessed chitosan-starch films blended with organic acids, showing complete degradation within 72–87 days, with increased plasticizer concentrations accelerating breakdown (Rachmawati et al., 2015). Starch-polyvinyl alcohol (PVA) blends also improved biodegradability, even with as little as 5 wt% starch. Dominici et al. (2020) demonstrated that thermoplastic starch combined with poly(butylene cyclohexane dicarboxylate) and 25 % adipic acid formed a fully bio-based material with superior flexibility, moisture resistance, and rapid biodegradability in composting conditions. Similarly, Ojogbo et al. (2020b) enhanced maize starch ester films with cellulose nanocrystals and montmorillonite organoclay, demonstrating increased biodegradability and accelerated weight loss with higher filler concentrations. These studies underscore the potential of starch-based biodegradable polymers to replace conventional plastics, offering an environmentally sustainable alternative that addresses decomposition challenges and resource conservation.

## 7 Food packaging Applications

### 7.1 Antibacterial activity

Natural antibacterial compounds are increasingly valued in food packaging for their ability to inhibit bacterial growth and extend shelf life. While starch lacks inherent antibacterial properties, combining it with bioactive agents transforms it into an effective active packaging material (Dai, Dong, et al., 2024; Zhu et al., 2023). Plant extracts rich in polyphenols, flavonoids, tannins, and alkaloids disrupt bacterial membranes, inhibit transport mechanisms, and block enzymatic functions (Manzoor et al., 2023). For example, incorporating pomegranate peel extract into starch matrices significantly inhibits *Staphylococcus aureus (*Khalid et al., 2018*).* Similarly, additional research has shown the antibacterial effectiveness of pomegranate peel extract when combined with different starches, including walnut shell cellulose and cashew nut-shell starch, against various bacterial strains (Harini et al., 2018). The film demonstrated inhibitory activity against *Listeria monocytogenes*, *Staphylococcus aureus*, *Escherichia coli*, *Pseudomonas aeruginosa*, *Klebsiella pneumoniae*, and *Salmonella enteritidis*, with inhibition zone diameters measuring 9.14 ± 0.11 mm, 4.25 ± 0.47 mm, 5.26 ± 0.71 mm, 2.38 ± 0.47 mm, 8.57 ± 0.61 mm, and 9.56 ± 0.22 mm, respectively (Harini et al., 2018). Other plant-based compounds, such as tea polyphenols and pitanga leaf extract, have also been integrated into starch-based films, enhancing their antibacterial properties (Sirisha Nallan Chakravartula et al., 2020). Tea polyphenols have demonstrated efficacy against *E. coli* and *Staphylococcus aureus*, whereas the combination of pitanga leaf extract and natamycin in starch/chitosan films has shown notable antioxidant and antifungal characteristics. Natural extracts like tea tree, cinnamon, and clove oils are frequently incorporated into starch-based films because of their inherent antibacterial qualities. For instance, tea tree oil films exhibited strong antibacterial activity against *E. coli* and *Staphylococcus aureus (*Feng et al., 2018*;* Zhang & Jiang*,* 2020*)*. Cinnamon oil and other essential oils have been incorporated into films made from starch and sodium bentonite clay nanoparticles, enhancing their efficacy against various bacteria. Clove oil demonstrated significant antimicrobial efficacy, exhibiting strong inhibitory effects against a broad spectrum of bacterial strains (Li et al., 2019). In agar diffusion assays, clove oil produced clear zones of inhibition ranging from 15 to 25 mm against common foodborne pathogens such as *Escherichia coli*, *Staphylococcus aureus*, and *Listeria monocytogenes*. Its potent activity is primarily attributed to the presence of eugenol, a major bioactive compound that disrupts microbial cell membranes and inhibits enzyme function (Lakshan et al., 2024). When incorporated into starch-based films, clove oil effectively reduces bacterial growth on food surfaces, resulting in extended shelf life and enhanced food safety. Comparative studies have shown that clove oil's antimicrobial performance is superior or comparable to other natural extracts like tea tree and cinnamon oils, making it a promising natural preservative agent for active packaging applications (Wang, Zhang, et al., 2021; Yang et al., 2018).

By-product extracts from food production, such as coffee grounds treated with citric acid, offer a cost-effective and sustainable means of improving starch films' antibacterial properties (Ounkaew et al., 2018). Such innovations make these films cost-effective and environmentally sustainable by recycling food industry waste. Using by-products can significantly reduce the cost of production and promote a circular economy in packaging materials. Numerous investigations have examined the integration of essential oils with by-product extracts to improve the antibacterial characteristics of starch-based films (Bae et al., 2022). For instance, films created using chitosan and starch, infused with pomegranate peel extract and thyme essential oil, effectively suppressed *Listeria monocytogenes* and prolonged the shelf life of beef (Mehdizadeh et al., 2020). In a similar vein, a film derived from cassava starch, infused with pumpkin residue extract and oregano essential oil, demonstrated significant antibacterial properties against *E. coli* and *Listeria monocytogenes* (dos Santos Caetano et al., 2018). Alongside essential oils, integrating nanomaterials like nano-silica (SiO2), nano‑titanium dioxide (TiO2), and nano-clay has significantly improved the antibacterial characteristics of starch-based films (Iamareerat et al., 2018). These nanomaterials exhibit exceptional surface activity and possess the ability to interfere with bacterial membranes. For instance, nano-SiO2 has proven effective in inhibiting *E. coli*, whereas nano-TiO2 integrated into starch/PVA films has exhibited improved antibacterial properties against *E. coli* and *Listeria monocytogenes* (Liu et al., 2019; Zhu et al., 2021). Metal nanoparticles such as silver (Ag) and zinc oxide (ZnO) enhance the antibacterial characteristics of these films, with Ag nanoparticles exhibiting a wide range of effectiveness (Khodaman et al., 2022; Kumar et al., 2021).

Chitosan, a biodegradable polysaccharide, strengthens starch-based films by interacting with bacterial cell walls, thereby extending the shelf life of foods such as tomatoes and pomegranates (Nair et al., 2020b). Additionally, natural antimicrobial agents like lysozyme and nisin enhance starch films' ability to inhibit bacterial growth, particularly against Gram-positive bacteria (Zhang & Rhim, 2022). The integration of plant extracts, essential oils, by-products, and nanomaterials into starch-based films presents a promising approach for developing sustainable, multifunctional food packaging. These advancements not only improve antibacterial properties but also promote eco-friendly solutions by utilizing natural and waste-derived materials (Ahari et al., 2025; Almajidi et al., 2024; Hong et al., 2022).

### 7.2 Antioxidant activity

Antioxidant activity is essential for improving the performance of starch-based films utilized in food packaging. Integrating antioxidant agents into these films enhances their oxidative properties, greatly prolonging the shelf life of packaged foods (Yu et al., 2025). Essential oils stand out for their impressive antioxidant properties and have been widely incorporated into starch-based films as a natural substitute for synthetic antioxidants. The molecules found in these essential oils can counteract detrimental free radicals like DPPH- and ABTS- +, thereby significantly lowering oxidative stress levels (Bibow & Oleszek, 2024). For example, Jamróz et al. (2018a) found that a film made from starch, furan cellulose, and gelatin containing 6 % tea tree essential oil demonstrated an antioxidant capacity of 985.7 ± 84.3 nmol/cm^2^. Moreover, the incorporation of lavender essential oil (Jamróz et al., 2018b) enhanced the antioxidant properties of starch-turbellarian-gelatin films by 88.97 % compared to the control films. Similarly, starch films made from foxtail millet and infused with clove leaf oil (Yang et al., 2018) exhibited notable DPPH and ABTS radical scavenging activities of 40.81 % and 70.59 %, respectively.

Polyphenols attract interest due to their antioxidant characteristics and are well-suited for improving starch-based films. Ellagic acid (EA), present in various fruits, has demonstrated remarkable capabilities in neutralizing free radicals when incorporated into apple starch films, enhancing oxidation resistance by as much as 7.1 times (Zhou et al., 2023). Curcumin, an essential component of turmeric, has been enhanced in chicken skin gelatin/rice starch composite films, reaching an outstanding 85.60 % DPPH antioxidant activity at a concentration of 0.03 g (Said & Sarbon, 2020). Menzel et al. (2020) also emphasized the beneficial antioxidant characteristics of starch films enhanced with phenolic compounds derived from rice straw. Integrating green tea extract into starch-based films significantly boosts antioxidant effectiveness. Research (Panrong et al., 2020)indicates that films made from a blend of thermoplastic starch and low-density polyethylene can effectively release green tea extract, achieving a rate of up to 70 % within ten hours in ethanol. Chollakup et al. (2020) noted that the release rates of polyphenols from cassava starch and whey protein blend films were greater in ethanol than in water. Estevez-Areco et al. (2020) created a double-layer film that integrates thermoplastic starch with ZnO nanorods and rosemary polyphenol-enriched PVA mats, showcasing strong antioxidant properties.

Natural antioxidants such as carotenoids and ascorbic acid demonstrate potential in starch-based films. de Oliveira Filho et al. (2024) integrated lycopene nanocapsules into tapioca starch to develop a biodegradable film that safeguards sunflower oil from oxidation. Kowalczyk et al. (2018); Kowalczyk et al. (2020) observed that incorporating ascorbic acid into oxidized potato starch films was directly linked to improved antioxidant activity. Incorporating essential oils, polyphenols, green tea extracts, carotenoids, and ascorbic acid into starch-based films greatly enhances their antioxidant capabilities, providing practical options for food preservation while delivering health advantages compared to synthetic alternatives. This advancement enhances food handling safety while meeting the increasing need for eco-friendly and sustainable packaging options within the culinary sector.

### 7.3 Anti-UV activity

Exposure to ultraviolet (UV) light readily produces free radicals, which can result in a decline in food quality due to lipid oxidation, nutrient loss, changes in pigment, and the emergence of unpleasant odors. Thus, it is essential to create films derived from starch that possess robust UV-blocking properties to preserve the integrity of food throughout storage and transportation (Cheng et al., 2024). Plant extracts, mainly those rich in polyphenols, offer a compelling solution because of their potent aromatic compounds that act as superior UV filters. For instance, Liu and Liu (2022) showed that adding eugenol acetate (EA) to starch films greatly enhanced light absorption throughout the UV–visible spectrum. At concentrations of merely 0.05 % and 0.1 % EA, these films effectively obstructed UV-A radiation (315–400 nm) almost entirely.

Furthermore, eugenol derived from clove leaf oil demonstrated maximum absorbance at 282 nm, successfully obstructing all UV radiation when used at concentrations of 0.5 %, 0.7 %, and 1 % (Tirado-Gallegos et al., 2018). Similarly, cassava starch films infused with rosemary extract showed minimal UV transmittance within the 200–400 nm spectrum, attributed to the UV-blocking aromatic compounds found in rosemary (Guz & Famá, 2024). Qin et al. (2020) incorporated betaine-rich red dragon fruit peel extract into starch/PVA films, producing a composite film that markedly decreased UV and visible light transmittance. The ability to block UV rays increased with higher concentrations of red dragon fruit peel extract, demonstrating its role in improving the light-blocking characteristics of starch/PVA films.

The incorporation of nano-fillers significantly improves the UV resistance of films made from starch. Zhang et al. (2018) discovered that incorporating nano-SiO2 into potato starch films reduced light transmittance and enhanced UV-blocking properties, thus providing superior protection against food deterioration caused by UV exposure. Wang et al. (2024) found that incorporating cellulose and starch crystals into hydroxypropyl high-amylose corn starch films markedly improved their durability against ultraviolet radiation. Babaei-Ghazvini et al. (2018) created a nanocomposite film using starch, kefiran, and ZnO that demonstrated enhanced UV protection, showing more excellent absorption in both UVC and UVA ranges as the ZnO content increased. These advancements in starch-based films are vital for protecting food from UV-induced damage, preserving active ingredients, reducing spoilage, and ensuring nutritional quality. Such films are particularly suitable for packaging items that do not require visual freshness assessments, including puffed foods, edible oils, and other sensitive products. Therefore, incorporating UV-resistant materials into food packaging is innovative and essential for prolonging shelf life and ensuring food safety.

### 7.4 Active packaging

A 3 % cassava starch coating (17–19 % amylose) combined with 0.05 % potassium sorbate has been shown to reduce respiration rate, improve water vapor permeability, and preserve the sensory quality of strawberries. Edible films are widely used in food packaging but can create conditions favorable for microbial spoilage over time (Rusli et al., 2024; Su et al., 2025). To counteract this, incorporating antimicrobial oils into starch-based films has become an effective strategy to extend shelf life and prevent contamination. Essential oils, known for their antimicrobial properties, have demonstrated significant efficacy in curbing microbial growth. For example, carvacrol-infused 2–3 % cassava starch coatings effectively inhibit pathogens on minimally processed pumpkins and papayas, reducing weight loss and delaying ripening (Chawla et al., 2021). Starch-based coatings are increasingly applied to nuts, baked goods, and fruits and vegetables. Rice starch films, offering greater flexibility than wheat or corn starch films, are particularly suitable for coating walnuts (Zuo et al., 2019). Combining 2 % glycerol with rice starch creates a uniform barrier against oxygen, moisture, and heat, significantly extending the shelf life of nuts (Sabaghi, 2024). These coatings also optimize storage efficiency by enabling husk and shell removal, reducing storage space requirements. Enhancing starch-based coatings with chitosan and red palm oil improves their smoothness and density, further enhancing protection (Maluin, 2024). Similarly, modifications to corn starch—such as incorporating ascorbic acid and tomato powder—have improved the shelf life, volume, and texture of bread made from frozen dough by preserving moisture and strengthening the gluten-starch network, likely due to increased glucose availability from acid hydrolysis (Ghoshal & Singh, 2024).

Recent advances underscore the potential of starch-derived films in food preservation. Devi et al. (2024) demonstrated that integrating nano-TiO2 into potato starch films enhances their mechanical strength, antibacterial properties, and barrier performance, making them ideal for packaging white mushrooms. The controlled release of antimicrobial compounds in starch-based films minimizes interactions with other food components while effectively reducing microbial contamination. Examples include grape seed extract-based intelligent packaging, which inhibits *Brochothrix thermosphacta*, and pomegranate peel particle-infused films, which curb *Staphylococcus aureus* and *Salmonella* (Andrade, 2023). Furthermore, phenolic-rich Viognier grape pomace, combined with cellulose nanocrystal-reinforced corn starch films, has been effective in inhibiting *S. aureus* and *Listeria monocytogenes* on deli meats (Ramachandraiah & Hong, 2022).

Advanced techniques have also led to the development of bilayer films with antimicrobial properties. For instance, heat-treated polyester films integrated with cassava starch effectively inhibit *Escherichia coli* and *Listeria innocua* due to carvacrol diffusion through the bilayer (Zhao et al., 2018). Additionally, nanocomposites combining eugenol with poly(3-hydroxybutyrate), maize starch, and montmorillonite effectively suppress *Botrytis cinerea* (Kerosenewala et al., 2023). Similarly, gelatin-starch nanocomposites enriched with nanocellulose and chitosan prevent fungal growth on pomegranate seeds.

Starch-based films also offer antioxidant protection. Packaging made from chitosan and thermoplastic corn starch inhibits yeast growth on bread, strawberries, and cheese, while the addition of rosemary nanoparticles enhances antioxidant activity in cassava starch films for controlled-release applications (Estevez-Areco et al., 2020). Pea and corn starch microparticles encapsulating quercetin exhibit improved heat stability and enhanced radical-scavenging activity when incorporated into films. Additionally, a bio-hybrid material composed of porous starch, halloysite nanotubes, and fucoxanthin demonstrated a controlled-release response to sunlight, reinforcing the potential of bio-composite films in antioxidant delivery (Sáez-Orviz et al., 2021; Trajkovska Petkoska et al., 2021). Edible films incorporating olive extracts have also been developed as cost-effective, eco-friendly packaging solutions that mitigate oxidative degradation in stored foods (Vianna et al., 2021; Wang et al., 2019; Yemenicioglu, 2022). These advancements highlight the growing potential of starch-based coatings and films as sustainable food preservation solutions, enhancing quality and safety.

### 7.5 Intelligent packaging

Bio-based smart packaging integrates sustainability with real-time food quality monitoring, ensuring health safety while offering economic and environmental benefits (Song et al., 2024). This packaging serves multiple functions—conveying, identifying, documenting, sensing, and tracking—providing essential insights to extend freshness, enhance safety, and indicate potential concerns. Natural components like plant extracts and dyes such as chlorophyll and carotenoids respond to pH changes, a reliable indicator of food quality (Shaikh et al., 2019). These bioactive compounds, essential for smart packaging films, often exhibit antimicrobial and antioxidant properties (Echegaray et al., 2024; Rezaei et al., 2024). Despite their dual role in active and intelligent packaging, research on their combined assessment remains limited. Integrating these features creates a biodegradable, multifunctional solution.

Starch-based smart packaging has shown significant potential as a visual freshness indicator. Choi et al. (2023) developed a colorimetric pH indicator film using agar, potato starch, and natural pigments from purple sweet potatoes. These films effectively detected pork spoilage by shifting from red to green. Similarly, Zhang et al., (2024) incorporated anthocyanins from purple sweet potatoes into carboxymethyl cellulose (CMC)/starch matrices, enabling real-time monitoring of raw grass carp freshness at 20 °C through color transitions from red to blue and green.

Further studies explored starch/polyvinyl alcohol (PVA) films infused with purple sweet potato extracts, offering both colorimetric indicators and antibacterial properties (Wang et al., 2018). Medina-Jaramillo et al. (2017) advanced this technology by incorporating green tea and basil extracts into cassava starch-glycerol films, where chlorophyll and carotenoids exhibited pH-responsive color changes, reinforcing their role in food quality assessment.

Expanding on this, Zhang, Huang, et al. (2020) demonstrated the effectiveness of anthocyanins from cabbage and sweet potato in starch-PVA films for real-time shrimp freshness assessment. These films exhibited distinct color changes in response to spoilage. (Yao et al., 2021) further enhanced packaging by integrating betacyanins from red pitaya, prickly pear, beetroot, globe amaranth flower, and red amaranth leaf into starch-PVA films, with shrimp spoilage indicated by a shift from pink to yellow (Mahin et al., 2025).

Recent innovations include chitosan-maize starch biopolymers infused with red cabbage anthocyanins for detecting fish fillet spoilage (Mileti et al., 2023). Cassava starch-based pH-sensitive films incorporating anthocyanins from pomace, blueberry residue, and *Lycium ruthenicum* have also been explored (da Costa et al., 2024). Notably, anthocyanins from grape skins, integrated into cassava starch sheets via extrusion, effectively tracked pH variations in beef and fish. Additionally, starch-PVA composite films infused with Roselle anthocyanins have shown promising results in assessing the freshness of raw silver carp (Shi et al., 2021).

## 8 Challenges in starch-based food packaging films

### 8.1 The complexities of starch and starch film processing

Starch, an abundant global resource, exhibits significant regional diversity, influencing the extensive research on starch-based films. While starches share a fundamental chemical structure, compositional differences profoundly affect film properties (Dai et al., 2019). For food packaging, starch-based films must possess superior mechanical properties. High tensile strength (TS) and elastic modulus (EM) ensure structural integrity, while adequate elongation at break (EAB) prevents failure during processing and transportation (Emblem & Emblem, 2012). Although impact strength and tear resistance are essential for commercial viability, they are often overlooked in early development. Barrier properties are equally critical, as low water vapor permeability (WVP) and oxygen permeability (OP) help preserve food quality. Transparency and aesthetic appeal enhance consumer acceptance, while additional factors such as UV transmittance, sensory attributes, toxicity, and environmental sustainability further determine a film's practicality (Gutiérrez et al., 2019; Osorio et al., 2019).

The amylose-to-amylopectin ratio is a key determinant of film properties. High-amylose starches, such as those from high-amylose corn, produce rigid films with increased tensile strength (TS) and elongation modulus (EM) but reduced elongation at break (EAB) due to enhanced crystallinity (Lu et al., 2019; Wang et al., 2018). These starches also exhibit lower water vapor permeability (WVP) and oxygen permeability (OP), improving barrier performance (Chinma et al., 2015); (Wang et al., 2018). However, an excessively low amylose content can impair film formation. Film characteristics are influenced by more than just amylose content. Factors such as amylose and amylopectin molecular weight, protein content, phosphate monoesters, granular morphology, and granule size distribution significantly impact mechanical properties (Baranzelli et al., 2019). Variations in plant cultivar and starch extraction timing further affect performance (Domene-López et al., 2019).

Retrogradation, an often-overlooked phenomenon, occurs when amylose molecules realign, expelling plasticizers and weakening film properties over time (Luo et al., 2023; Tao et al., 2024). To counteract this, researchers incorporate plasticizers or additives like carbohydrates, proteins, and salts, though these can compromise mechanical properties and raise costs (Cheng et al., 2019). Recent studies explore novel plasticizers and mixed systems to inhibit retrogradation, yet further research is needed to fully understand its long-term effects on film durability (H. Zhang et al., 2020; W. Zhang et al., 2020). Advancing starch-based film technology requires a deeper understanding of these complex interactions to enhance durability, functionality, and commercial viability.

### 8.2 Hurdles to commercialization of starch films for packaging applications

Plastics have become a cornerstone of packaging solutions due to their lightweight nature, cost-effectiveness, and ease of large-scale commercial processing. Among the most common methods, plastic films are thermally processed using advanced extrusion technologies, offering high efficiency and scalability (Prabha et al., 2021). In stark contrast, starch-based films' mechanical and thermal processing presents significant challenges. Starch requires abundant processing solvents and is prone to degradation under mechanical and thermal stresses, rendering it unsuitable for conventional extrusion methods.

However, innovation in starch-containing resin formulations has opened the door to practical applications. A prime example is BioBag®, a fully compostable solution crafted from plant- and oil-based materials integrated with synthetic, compostable polymers (Muiruri et al., 2022). These advances illustrate the potential of sustainable materials to meet commercial demands. Yet, like any pioneering sustainable technology, these products face performance gaps compared to their petroleum-derived counterparts. Bridging these gaps requires targeted improvements in material properties and the development of technologies that further enhance sustainability (Saberi Riseh et al., 2023).

### 8.3 Compliance with FDA/EFSA standards

While starch-based films offer promising biodegradable alternatives for food packaging, ensuring their compliance with stringent regulatory standards set by authorities like the U.S. Food and Drug Administration (FDA) and the European Food Safety Authority (EFSA) presents notable challenges (Kola & Carvalho, 2023). These agencies mandate rigorous assessments of material safety, migration limits of additives or nanoparticles, and potential toxicological effects on food products. Incorporating additives such as nanoparticles (e.g., TiO₂, SiO₂) to enhance UV protection and mechanical properties raises concerns about nanoparticle migration and consumer exposure (Dhalsamant et al., 2025; Prajapati, 2022). Currently, regulatory frameworks for nanoparticles in food contact materials are still evolving, requiring comprehensive toxicological data and standardized testing protocols. Moreover, variability in starch sources, chemical modifications, and plasticizer types can affect film composition consistency, complicating compliance verification. The hydrophilic nature of starch films also increases susceptibility to moisture absorption, potentially altering film integrity and increasing migration risks (Peerzada Gh et al., 2023). Additionally, ensuring the films meet food-grade standards without compromising biodegradability or functional performance remains a critical balance (Vilpoux, 2023). Addressing these regulatory and safety challenges demands multidisciplinary research integrating material science, toxicology, and regulatory expertise to develop starch-based films that are not only effective but also safe and compliant for widespread food packaging applications.

## 9 Conclusion

Starch-based edible packaging films represent a transformative shift toward sustainable food packaging, offering a biodegradable, low-cost, and renewable alternative to petroleum-based plastics. Their versatility, transparency, and barrier properties have made them attractive candidates in addressing the mounting environmental burden of plastic waste. Currently, starch films are successfully applied in niche packaging formats such as single-use wraps, pouches, and coating layers for fruits, vegetables, or snacks. Their compatibility with food contact standards, odorless and tasteless nature, and low oxygen permeability further support these applications. However, challenges such as high water sensitivity, limited mechanical strength, and poor long-term stability still restrict their broader industrial adoption. To overcome these limitations, significant advancements are being explored. Nanotechnology has played a central role in improving film properties—incorporating nanoclays, nanocellulose, titanium dioxide, or silicon dioxide enhances mechanical strength and UV resistance while reducing water permeability. Similarly, integrating bioactive compounds like clove or tea tree oil improves antimicrobial functionality, extending the shelf life of perishable products. Moreover, smart packaging features—such as pH-sensitive color indicators embedded into starch matrices—are enabling real-time food freshness monitoring, representing a leap from passive to intelligent packaging systems. Despite these innovations, most starch-based packaging remains at a laboratory or pilot scale. Widespread market adoption depends heavily on economic feasibility, manufacturing scalability, and compliance with international food safety regulations, including those set by the FDA and EFSA. For example, standardization in processing parameters and migration testing for bioactive additives or nanomaterials is essential to ensure consumer safety and regulatory approval. Currently, only a handful of starch-based films have undergone rigorous regulatory review, limiting their entry into mainstream packaging supply chains.

A critical step toward industrial viability is conducting robust Life Cycle Assessment (LCA) to evaluate the environmental footprint of starch films across production, usage, and disposal stages. Studies have shown that starch-based films typically exhibit lower greenhouse gas emissions and energy consumption compared to conventional plastics when derived from agricultural waste or by-products. However, the energy and water inputs required for starch extraction, plasticization, and film casting must be optimized to realize true sustainability benefits. Integrating LCA into early design stages ensures a data-driven pathway to reduce environmental impact while maximizing resource efficiency.

From a market perspective, starch-based films are gaining momentum, especially in regions with strict plastic bans and growing consumer demand for eco-friendly alternatives. The global biodegradable packaging market is projected to exceed $120 billion by 2032, with starch-based solutions accounting for a significant share. Nonetheless, their uptake is largely confined to premium and health-conscious product segments due to current cost and performance limitations. Looking ahead, the distinction between current capabilities and future potential is critical. Today, starch films perform effectively in dry food packaging and as active coatings. In the future, developments in 3D printing, hybrid polymer blending, and biotechnology will allow tailored film structures with enhanced moisture resistance, thermal stability, and intelligent behavior. Furthermore, advancements in circular bioeconomy practices—such as sourcing starch from agricultural residues and enabling compostability—will amplify environmental benefits. Starch-based edible films have transitioned from conceptual innovations to functional solutions in sustainable food packaging. While current technologies meet select application demands, future progress hinges on scaling up production, refining materials for diverse food environments, and ensuring full regulatory compliance. Through continued interdisciplinary collaboration, starch-based packaging is well-positioned to become a cornerstone in advancing global sustainability and circular economy goals.

## Consent of participate

Not applicable.

## CRediT authorship contribution statement

**Arun Karnwal:** Writing – review & editing, Writing – original draft, Supervision, Resources, Investigation, Conceptualization. **Abdur Rauf:** Writing – review & editing, Visualization, Validation, Resources, Formal analysis. **Amar Yasser Jassim:** Writing – review & editing, Visualization, Validation, Resources, Formal analysis, Data curation. **Manickam Selvaraj:** Writing – review & editing, Validation, Software, Resources, Formal analysis. **Abdel Rahman Mohammad Said Al-Tawaha:** Writing – review & editing, Visualization, Investigation, Formal analysis, Data curation. **Piyush Kashyap:** Writing – review & editing, Visualization, Validation, Formal analysis, Data curation. **Deepak Kumar:** Writing – review & editing, Validation, Resources, Methodology, Formal analysis, Data curation. **Tabarak Malik:** Writing – review & editing, Writing – original draft, Visualization, Validation, Formal analysis, Data curation.

## Consent to publish

Not applicable.

## Ethical approval

Not applicable.

## Funding

No funds or grants are received.

## Declaration of competing interest

The authors declare that they have no known competing financial interests or personal relationships that could have appeared to influence the work reported in this paper.

## Data Availability

No data was used for the research described in the article.

## Appendix A Supplementary data

Supplementary material Image 1
