# Supplementary material for: Advanced starch-based films for food packaging: Innovations in sustainability and functional properties
Source: Food Chem X. 2025 Jun 17;29:102662. doi: 10.1016/j.fochx.2025.102662 (PMC12226126; doi:10.1016/j.fochx.2025.102662)
Supplement: Supplementary file 1 — Supplementary material [file mmc1.docx]

| **Supplementary table 1**: Comparison of Starch Film Preparation Methods: Food Materials, Starch Types, and Key Considerations (Dai et al., 2019; do Val Siqueira et al., 2021; Said & Sarbon, 2020) | | | | |
| --- | --- | --- | --- | --- |
| **Method of Starch Film Preparation** | **Type of Food Used** | **Starch Type** | **Major Concerns** | **Examples** |
| **Solution Casting** | - Fruit juices (apple, orange)  - Dairy products (milk, yogurt)  - Edible coatings | - Corn starch  - Potato starch | - Control of humidity during drying.  - Consistency in film thickness.  - Drying time and temperature. | - Starch-based films for fruit coatings.  - Dairy-based edible films for yogurt containers. |
| **Extrusion Process** | - Bakery products  - Snacks (chips, crisps)  - Pasta | - Maize starch  - Wheat starch | - High temperature and shear forces may degrade starch.  - Controlling film thickness and density. | - Starch-based snacks with an extruded edible film for preservation.  - Extruded pasta with a starch film to retain moisture. |
| **Electrospinning** | - Food packaging films  - Dairy products packaging | - Potato starch  - Rice starch | - Nanofiber formation may affect the mechanical properties.  - Difficulty in achieving uniform thickness. | - Nano-starch films for packaging dairy products.  - Electrospun starch films for food packaging with antimicrobial properties. |
| **3D-printing** | - Customized food shapes  - Cake decoration | - Modified corn starch  - Tapioca starch | - Limited by printer resolution and material behavior.  - Complex printing patterns require precise starch formulations. | - 3D-printed edible food packaging.  - Customized 3D-printed cake decorations using starch-based inks. |
| **Reactive Extrusion** | - Frozen foods (vegetables, meats)  - Bakery products | - Corn starch  - Potato starch | - Excessive heating can break down starch polymers.  - Potential for reduced mechanical strength of films. | - Starch-based films for frozen food packaging.  - Bakery films used to encapsulate fillings in pastries. |
| **Nanotechnology** | - Nutrient delivery systems  - Functional food packaging | - Corn starch  - Tapioca starch | - Difficulty in controlling nanoparticle size.  - Stability of nanoparticles in the final product. | - Starch nanoparticles for controlled release of nutrients.  - Nano-starch films for enhanced food packaging properties. |
| **Blow Moulding Method** | - Beverage containers  - Bottled sauces | - Modified starch  - High amylose starch | - Film transparency issues.  - Difficulties in uniform wall thickness during molding. | - Starch-based biodegradable bottles for beverages.  - Blow-molded starch packaging for sauces. |

| **Supplementary table 2:** Comparison of Properties, Influencing Factors, and Applications of Starch-Based Films in Packaging (Jiang et al., 2020; Li et al., 2019; Lim et al., 2020; Saliu et al., 2019; Wang et al., 2024; Zhu et al., 2023; Zuo et al., 2019) | | | | |
| --- | --- | --- | --- | --- |
| **Property** | **Description** | **Factors Influencing** | **Comparison with Other Materials** | **Applications** |
| **Water Vapor Permeability (WVP)** | Measures the rate of water vapor passing through the film. | - Film composition (amylose vs. amylopectin ratio) - Plasticizers (e.g., glycerol, sorbitol) - Relative humidity - Film thickness | Higher than synthetic polymers like polyethylene (PE) but lower when blended with hydrophobic additives (e.g., waxes) | Food packaging for dry products where water vapor resistance is less critical. |
| **Gas Permeability** | Includes permeability to gases like oxygen (O₂) and carbon dioxide (CO₂). | - Crystallinity of starch - Crosslinking agents - Additives such as nanofillers or lipids | Lower permeability to oxygen than PE but higher compared to polyethylene terephthalate (PET). | Packaging of fresh produce to allow moderate gas exchange and extend shelf life. |
| **Oil and Grease Resistance** | Resistance to oil and grease migration through the film. | - Incorporation of hydrophobic components - Coating with lipids or waxes | Moderate resistance; improved when layered or coated with hydrophobic materials. | Wrapping fatty foods, e.g., butter, cheese, or snacks. |
| **Moisture Absorption** | Tendency to absorb moisture from the environment, which can affect mechanical and barrier properties. | - Hygroscopic nature of starch - Degree of film gelatinization - Plasticizer concentration | Higher moisture absorption compared to synthetic polymers; can be reduced by blending with hydrophobic polymers. | Suitable for applications in controlled humidity conditions or combined with moisture-resistant layers. |
| **Transparency** | Clarity of the film, which is critical for visual inspection of packaged goods. | - Homogeneity of the starch matrix - Type of starch used (native or modified) - Additives | Comparable to synthetic polymers like low-density polyethylene (LDPE) in clarity but depends on composition. | Used for transparent packaging of food items such as fruits, vegetables, and baked goods. |
| **Solubility in Water** | Degree to which the film dissolves in water. | - Amylose content (higher reduces solubility) - Crosslinking density - Inclusion of hydrophobic agents | Dissolves more readily than synthetic films unless treated or blended with insoluble agents. | Edible films for food packaging or single-use dissolvable pouches for pre-measured ingredients (e.g., detergents). |
| **Mechanical Durability in Humid Environments** | Resistance to mechanical property degradation under high humidity. | - Plasticizer type and concentration - Crosslinking agents - Incorporation of nanoparticles or fibers | Degrades more rapidly than synthetic polymers under humid conditions, limiting use in wet environments. | Ideal for applications in dry or moderately humid conditions, such as dry food packaging. |
| **Resistance to Microbial Growth** | Film's ability to resist microbial degradation. | - Type of starch (native or modified) - Presence of antimicrobial agents (e.g., essential oils, silver nanoparticles) | Lower resistance compared to synthetic materials unless fortified with antimicrobial additives. | Used in active packaging systems that incorporate antimicrobial agents to enhance shelf life. |
| **Thermal Stability** | Stability of the film under varying temperatures. | - Thermal treatment during preparation - Addition of stabilizers or nanofillers | Thermal stability is lower than synthetic polymers, but blending with additives improves it significantly. | Used in temperature-sensitive packaging where exposure to high heat is minimal. |
| **Resistance to UV Radiation** | Ability to block or allow UV light transmission through the film. | - Presence of UV-blocking agents (e.g., titanium dioxide, zinc oxide) - Modification with pigments or fillers | Lower UV resistance compared to synthetic polymers unless additives are used. | Used for products requiring UV protection, such as certain pharmaceuticals and light-sensitive food products. |

| 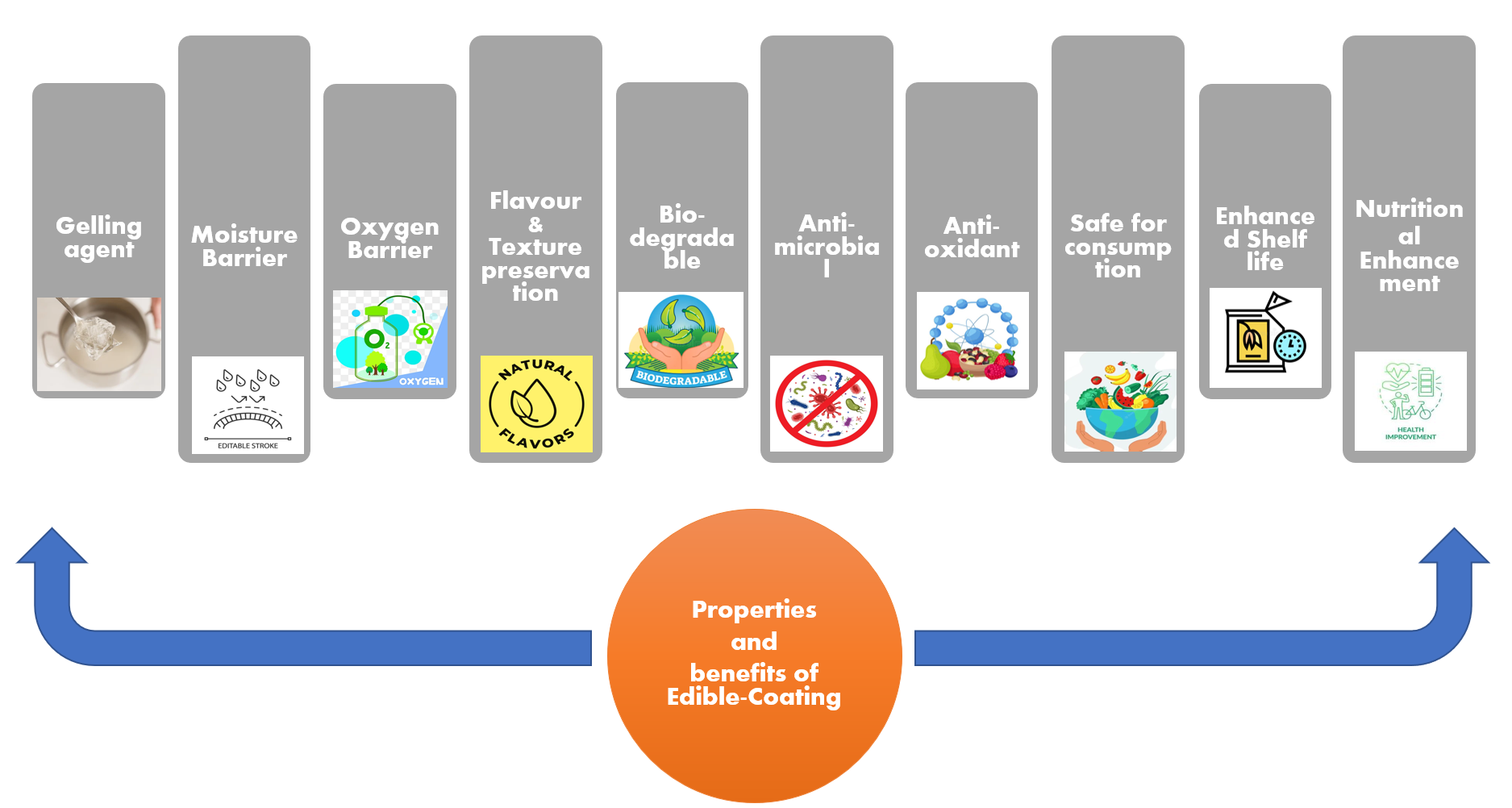 |
| --- |
| **Supplementary Figure 1**: Beneficial properties and advantages of edible food coatings in food. |
